# Explainable machine learning to predict the cost of capital

**DOI:** 10.3389/frai.2025.1578190

**Published:** 2025-04-10

**Authors:** Niklas Bussmann, Paolo Giudici, Alessandra Tanda, Ellen Pei-Yi Yu

**Affiliations:** ^1^Department of Economics and Management, University of Pavia, Pavia, Italy; ^2^CAM-Risk - Centre for the Analysis and Measurement of Global Risks, University of Pavia, Pavia, Italy; ^3^Department of Management, Birkbeck College, University of London, London, United Kingdom

**Keywords:** explainable AI, Shapley Values, XGBoost models, cost of capital, non-financial disclosure

## Abstract

This study investigates the impact of financial and non-financial factors on a firm's ex-ante cost of capital, which is the reflection of investors' perception on a firm's riskiness. Departing from previous literature, we apply the XGBoost algorithm and two explainable Artificial Intelligence methods, namely the Shapley value approach and Lorenz Model Selection to a sample of more than 1,400 listed companies worldwide. Results confirm the relevance of key financial indicators such as firm size, ROE, firm portfolio risk, but also individuate firm's non-financial features and country's institutional quality as relevant predictors for the cost of capital. These results suggest the importance of non-financial indicators and country institutional quality on the firm's ex-ante cost of equity that expresses investors' risk perception. Our findings pave the way for future investigations on the impact of ESG and country factors in predicting the cost of capital.

## 1 Introduction

The employment of artificial intelligence (AI) tools in finance is becoming quite common: by levering on multidimensional and high-frequency data, AI tools can overcome stringent assumption on the distribution of variables and on the linear relationships between dependent and independent variables. Hence, they contribute to the more accurate prediction of returns and risk of securities and in risk management (Cao, 2022; Lin, 2018; Liu et al., 2022; Ortmann, 2016; Simonian, 2019). Despite the advantages of AI tools, these can also be very opaque, making the economic and financial interpretation of results of the algorithm very difficult for an investor. Additionally, regulators have warned investment firms and financial institutions against the use of AI tools, and the interpretability and accountability of financial models used to determine investors' and intermediaries' choices is key in the policymakers' agenda (Weber et al., 2024).

One way to address this issue is to employ the so-called Explainable AI (or XAI) methods, that are able to “open” the black box and allow to interpret results and individuate their drivers. Among XAI methods, the Shapley Values or the SHapley Additive exPlanations (SHAP) Framework has been recently employed also in the corporate finance and banking literature (Kumar et al., 2020; Fryer et al., 2021; Bitetto et al., 2023; Shalit, 2023; Basher and Sadorsky, 2025). Recent reviews argue that XAI is becoming increasingly employed in the finance literature, especially in the area of credit management, stock price predictions, and fraud detection (Černevičienė and and Kabašinskas, 2024; Weber et al., 2024). Nevertheless, to the best of our knowledge, XAI has not yet been employed in the prediction of the cost of capital.

Within this framework, this paper is the first to apply XAI tools to estimate the cost of capital for a sample of large listed companies. The cost of capital represents the remuneration investors require to provide funds to a firm and it is determined by a company's financial and non-financial characteristics as well as country specific features. Previous studies choose between two main approaches to proxy the cost of capital: a historical approach (ex-post) or an implied (ex-ante) approach. The first approach is suitable for finding the determinants of the historical cost of capital (e.g., Weighted Average Cost of Capital—WACC or Capital Asset Pricing Model—CAPM) (Wong et al., 2021; Desender et al., 2020; Shad et al., 2020). The second approach, based on the ex-ante or implied cost of capital, interprets the cost of capital as the risk associated to an investment in the company by an investor (Hail and Leuz, 2006; Pástor et al., 2008). Studies taking this second approach often employ Price Earning Growth (PEG) models. These rely on analysts' forecasts for future earnings to predict the cost of capital as the implied return on the company's equity investment (Gupta, 2018; Garćıa-Sánchez et al., 2021; Yu et al., 2021).

In the literature, a company's cost of capital is generally determined by internal firm financial characteristics, market features and, less often, country characteristics (Breuer et al., 2018; Desender et al., 2020; Wang et al., 2021; Yu et al., 2021). Financial characteristics generally include size, economic and operating performance measures, leverage, working capital, investments in research and development and intangibles (Zimon et al., 2024; Houqe et al., 2024).

Recent studies include the non-financial behaviour of companies among the determinants of the cost of capital (El Ghoul et al., 2011; Dorfleitner et al., 2015). ESG performance can, in fact, determine companies' riskiness and value, influencing future revenues and earnings (D'Amato et al., 2017; Global Sustainable Investment Alliance, 2018; Widyawati, 2020; Yu et al., 2021).

The literature elaborated and discussed several theories to understand the importance of corporate responsibility or sustainable behaviour. The traditional shareholder theory (Ross, 1973; Jensen and Meckling, 1976) posits that the only objective of companies is to maximise value for shareholders, while alternative theories (e.g., the stakeholder theory) welcome the opportunity to include all stakeholders' interests, in line with the long term objective of value creation (Garriga and Melé, 2004). Additionally, sustainable practices can constitute a competitive advantage for companies (Sharma and Vredenburg, 1998; Campbell, 2007; Surroca et al., 2010). Reducing the intensity of carbon emissions (or GHG emissions) and the ability to adopt more sustainable practices, both for the environmental domain and the social dimension, generally reduces the cost of capital (Bui et al., 2020; Yu et al., 2021; Barg et al., 2024; Feng and Wareewanich, 2024). Adopting “good governance” practices also signals reduced riskiness to the market. For instance, gender representation and the presence of independent directors decrease the cost of capital (Tran, 2020; Huang et al., 2021; Sarang et al., 2024a,b). Finally, managerial ability can influence the firms' ability to generate revenues and, in last instance, the cost of funding (Dalwai et al., 2023).

In addition, the institutional quality of countries where firms are located can affect the perceived riskiness of their business and, as a result, the cost of capital. Previous empirical literature has employed different measures to capture countries' features and finds that institutional quality reduces the cost of equity. For instance, Eldomiaty et al. (2016) employ the Economic Freedom Indicator, while Grira et al. (2019) employ the measures developed by the International Country Risk Guide on the quality of institutions, democratic tendencies, corruption, and government action. A paper by Banerjee et al. (2022) finds that the level of corruption influences the cost of capital when policy uncertainty is high. More recently, Nasrallah et al. (2025) find that country-level governance has a negative relationship with the cost of equity.

In this paper, we employ the World Bank's Worldwide Governance Indicators (World Bank, 2018) and the Human Development Index (UNDP, 2018) as proxy of the country's non-financial characteristics that are able to influence the cost of capital of listed companies.

With reference to the methodological aspects, past literature on the cost of capital employs linear models to investigate the impact of financial and non-financial characteristics on the cost of capital. However, some studies allow non-linear relationships between dependent and independent variables, for instance by introducing the square of independent variables. Among the studies that posit non-linear effect of non-financial variables, Yu et al. (2021) control the effect of environmental disclosure on the cost of equity and also use the square of environmental disclosure to argue that, over a certain threshold, additional environmental disclosure can curb the positive effect of the variable on the cost of equity.

To overcome this limitation, this paper applies the XGBoost algorithm and two explainable AI methods, Shapley Values and Lorenz Zonoids, to detect which financial and non-financial factors are good candidates as predictors of the cost of capital of more than 1,400 multinational companies listed in 43 different countries for the period 2013–2019.

Thanks to our approach we are able to provide an intuitive explanation of the contribution of each variable included in the analysis to the model prediction, thereby “opening” the black-box of the AI methodology. We contribute to the literature by determining the most relevant financial and non-financial features that predict the implied cost of capital, without making any a priori assumption on the relationships between them and investigating the role of financial and non-financial features both at firm and country levels. We find that besides the traditional drivers of cost of capital—i.e., size, profitability and liquidity—non-financial features of companies and countries are able to drive the prediction of the cost of capital. Emission intensity is found to predict a higher cost of capital, suggesting the investors request higher return from companies with high emissions. But companies located in countries with good institutional quality benefit from a lower cost of capital.

Our results have important managerial implications: on one hand, investors can use our results to choose the portfolio allocation that best aligns with their preferences and, on the other hand, companies can have a better understanding of how to improve their financial and non-financial indicators to access cheaper funding. Additionally, the results support the policymakers' initiatives aimed at improving corporate disclosure and performance in the non-financial indicators and can support policies to improve the institutional quality of the country, to attract investors and allow firms to collect the necessary resources to fund their investments, also in the pursuit of a more sustainable production system and economic system. The remainder of the paper is organised as follows: Section 2 introduces the methodology; Section 3 describes the data and the variables employed; Section 4 presents the empirical findings; Section 5 discusses our results and, finally, Section 6 concludes.

## 2 Methodology

To analyse the data set and predict the cost of capital, we use the well-known extreme gradient boosting machine learning model (XGBoost) (Chen and Guestrin, 2016; Bentéjac et al., 2021). XGBoost is an ensemble learning method that is particularly well-suited to large structured data sets. It is a supervised machine learning model that combines decision tree models with gradient boosting. The model applies decision trees, which are weak classifiers, to a data set, where each subsequent decision tree is built to correct the errors of the previous tree model (e.g., Chen and Guestrin, 2016). The XGBoost model is a black-box model: its predictions are not explained in terms of their drivers. However, as shown in several papers, different explainable AI (XAI) methodologies can be applied to explain the predictions of Machine Learning models and hence “open” the black box (Bussmann et al., 2020, 2021; Gramegna and Giudici, 2021, 2022; Lundberg et al., 2020; Adam, 2024; Audemard et al., 2024).

The application of these methods is becoming more common also in corporate finance (Ghoddusi et al., 2019). Recently Lin and Bai (2022) apply a machine learning approach to estimate the determinants of the cost of debt for 40 listed companies in the mining, steel, and power industries. Tron et al. (2023) investigate the ability of corporate governance features of non-listed companies to determine corporate defaults. Other contributions study AI in the risk management in finance (Gan et al., 2020) or the application of AI to corporate financial functions (e.g., Polak et al., 2020). Černevičienė and and Kabašinskas (2024) find that AI is heavily employed in studies on credit risk determination, stock price predictions, and fraud detection. Kumar et al. (2025) provide a systematic review of papers employing AI in the field of Fintech and find some areas are well explored (namely, risk management, portfolio optimisation, and applications related to the stock market), while others remain understudied. In this paper, by applying different XAI methods to our XGBoost model we aim to identify which financial and non-financial variables mostly affect the prediction of the cost of capital.

To produce a ranking of the variables, the XGBoost Python package includes an integrated feature importance plot function. The algorithm measures how often each variable is used to split the data, across all decision trees. With this technique, variables that are often used for important splits are identified as the most important for the model predictions (Chen and Guestrin, 2016).

Another popular method to explain complex ML models is the SHapley Additive exPlanation (SHAP) framework. The SHAP framework defines an interpretation for each prediction in the form of an explanation model. It calculates the average marginal contribution of each feature to the predictions across all possible feature combinations (Lundberg and Lee, 2017). The underlying Shapley values method (Shapley, 1953) belongs to the class of additive feature attribution methods and derives from cooperative game theory.

The SHAP algorithm calculates Shapley values, which characterise predictions as linear combinations of binary variables, indicating whether or not each variable is included in the model. As a result, a SHAP value is calculated for each variable, representing the relative contribution to the model predictions (Lundberg and Lee, 2017). The explanation model is a linear function of the binary variables and is defined as in Equation 1.


(1) $$\begin{array}{l} g\left(x^{\prime }\right)=\phi_{0}+\overset{M}{\underset{i=1}{\sum }}\phi_{i}x_{i}^{\prime }. \end{array}$$

where:

- *x*′ ∈ {0, 1}^*M*^,
- ϕ_*i*_ ∈,
- *M* is the number of independent variables (Lundberg et al., 2020).

The Shapley value approach, underlying the SHAP algorithm, belong to the class of additive feature attribution methods. Indeed, Lundberg and Lee (2017) showed that the Shapley value method is the only explanation model that jointly satisfies the characteristics of local accuracy, missingness and consistency. Local accuracy indicates that the sum of all variables of the explanation model approximates the output of the original model. Missingness denotes that missing variables do not receive any importance in the explanation model. Consistency states that a change in the model, which leads to an increase in the contribution of a variable, cannot decrease its importance (Lundberg et al., 2020).

The above characteristics are achieved by assigning to each feature vector, a feature attribution value, which is defined as follows (Equation 2). The *i*-th Shapley value of a variable *X*_*k*_, (*k* = 1, …, *K*) is:


(2) $$\phi ({\hat{f}}^{k}(X_{i}))=\sum_{X^{\prime }\subseteq C(X)\backslash X_{k}}\frac{\left|X^{\prime }\right|!\left(K-\left|X^{\prime }\right|-1\right)!}{K!}[\hat{f}{\left(X^{\prime }\cup X_{k}\right)}_{i}-\hat{f}{\left(X^{\prime }\right)}_{i}],$$

where $C\left(X\right)\backslash X_{k}$ is the set of all the possible model configurations which can be obtained excluding variable *X*_*k*_; $\hat{f}{\left(X^{\prime }\cup X_{k}\right)}_{i}$ and $\hat{f}{\left(X^{\prime }\right)}_{i}$) are the predictions obtained including and excluding variable *X*_*k*_.

The Shapley contribution of *X*_*k*_ is the sum (or the mean) of all Shapley values (Lundberg et al., 2020). Although Shapley values are widely used in the recent machine learning literature, they have a drawback: their values are not normalised and, therefore, cannot be easily interpreted and compared across different applications.

To overcome this issue, we employ the Lorenz Model Selection approach introduced by Giudici and Raffinetti (2020) to perform variable selection and simplify the machine learning model. The underlying Lorenz Zonoid approach is based on the research of Koshevoy (1995) for empirical distributions and on Mosler (1994) for general probability distributions.

Lorenz Model Selection offers a novel method to select variables not on the basis of correlation, but on the basis of a mutual notion of variability. This makes them more robust to outliers (Babaei et al., 2025). In the univariate case, the Lorenz Zonoid values equate to the Gini coefficient, which can be used to measure the contribution of each explanatory variable to the predictive power of a linear model more accurately. As shown by Lerman and Yitzhaki (1984) in the univariate case, the Lorenz Zonoid *LZ*_*d* = 1_ can be expressed by the formula in Equation 3


(3) $$\begin{array}{l} LZ_{d=1}\left(Y\right)=\frac{2Cov\left(Y,r\left(Y\right)\right)}{\mu }. \end{array}$$

where:

- *Y* is the dependent variable,
- μ is the mean value of Y, and
- *r*(*Y*) is the rank score of Y variables.

Giudici and Raffinetti (2020) show that if we consider the dependent variable *Y* and the independent variables *X*_1_, ..., *X*_*h*_, ..., *X*_*k*_ with *h* = 1, ..., *k*, and we apply a model on this data set, we receive the predictions Ŷ_*X*_1_, ..., *X*_*k*__. The Lorenz Zonoid values are defined accordingly as in Equation 4 and Equation 5.


(4) $$\begin{array}{l} LZ_{d=1}\left(Y\right)=\frac{2Cov\left(Y,r\left(Y\right)\right)}{n\mu } \end{array}$$

and


(5) $$\begin{array}{l} LZ_{d=1}\left({Ŷ}_{X_{1},...,X_{k}}\right)=\frac{2Cov\left({Ŷ}_{X_{1},...,X_{k}},r\left({Ŷ}_{X_{1},...,X_{k}}\right)\right)}{n\mu }. \end{array}$$

where:

- *n* is the number of all observations,
- *r*(Ŷ_*X*_1_, …*X*_*k*__) is the rank score of the predicted variables Ŷ_*X*_1_, ..., *X*_*k*__.

The formulae described above can be rearranged in such a way that the underlying model predictions are generalised and rearranged in a non-decreasing manner, thus yielding a measure of marginal dependence, called the Marginal Gini Coefficient (MGC), which determines the explanatory power of each variable. The *MGC* can be calculated with the following Equation 6, for any variable *X*_*h*_, (*h* = 1, ..., *k*).


(6) $$MGC(Y|X_{h})=\frac{LZ_{d=1}({\hat{Y}}_{X_{h}})}{LZ_{d=1}(Y)}=\frac{Cov({\hat{Y}}_{X_{h}},r({\hat{Y}}_{X_{h}})}{Cov(Y,r(Y))}.$$

The previous formulae can also be rearranged to calculate the additional (partial) contribution of a new explanatory variable, *X*_*k*_+1, to an existing model, resulting in the partial Gini coefficient (PGC) (Equation 7).


(7) $$\begin{array}{l} PGC\left(Y,X_{k}+1|X_{1},...,X_{k}\right)=\frac{LZ_{d=1}\left({Ŷ}_{X_{1},...,X_{k}+1}\right)-LZ_{d=1}\left({Ŷ}_{X_{1},...,X_{k}}\right)}{LZ_{d=1}\left(Y\right)-LZ_{d=1}\left({Ŷ}_{X_{1},...,X_{k}}\right)}. \end{array}$$

We employ the PGC to measure the contribution of each additional variable to the predictive accuracy of our model, within a stepwise model selection procedure.

In order to compare any two models, we need to define the payoff. To do this, we calculate the following difference for any statistical unit *i*, reported in Equation 8:


(8) $$\begin{array}{l} Poff\left(X_{i}^{k}\right)=\hat{f}{\left(X\cup X_{k}\right)}_{i}-\hat{f}{\left(X\right)}_{i}, \end{array}$$

where:

- $\hat{f}{\left(X\right)}_{i}$ represents the predictions of a model and
- $\hat{f}{\left(X\cup X_{k}\right)}_{i})$ represents the predictions of a model after including an additional independent variable.

If we replace the model predictions with the *PGC*, we receive for a given set of statistical units the following Equation 9:


(9) $$\begin{array}{l} Poff\left(X^{k}\right)=LZ_{d=1}\left({Ŷ}_{X_{1},...,X_{k}}\right)-LZ_{d=1}\left({Ŷ}_{X_{1},...,X_{k-1}}\right), \end{array}$$

where:

- *LZ*_*d* = 1_(Ŷ_*X*_1_, ..., *X*_*k*−1__) represent the Lorenz Zonoid values of a model and
- *LZ*_*d* = 1_(Ŷ_*X*_1_, ..., *X*_*k*__) represent the predictions of a model after including an additional independent variable.

Once calculated, the pay-off can be assessed in terms of statistical significance, by means of an appropriate test that compares the predictive accuracy of the two models being compared.

As the cost of capital is a continuous variable, we propose to employ the Diebold Mariano test (Diebold and Mariano, 2002), which compares the forecasting accuracy of a continuous response by two competing models.

To perform the test the model predictions need to be compared with the actual observations, and forecast errors calculated. The null hypothesis of the test states that the forecast errors of any two models do not show statistically significant differences and thus the models being compared could not be identified as statistically significant different in terms of their predictive accuracy.

The null hypotheses of null difference between the forecast errors is defined by *E*[*g*(*e*_*i*_*t*)], or *E*[*d*_*t*_] = 0, where *g*(*e*_*i*_*t*) is a function of the forecast error and *d*_*t*_ = [*g*(*e*_*i*_*t*)−*g*(*e*_*j*_*t*)] is the loss difference. In other words, the null hypothesis that the predictive accuracy of both models is equal can also be expressed as a null hypothesis that the difference between the population mean of the losses is equal to zero.

To determine whether the difference is statistically significant or not, the test statistic can be compared to a critical value from an appropriate distribution, whose parametric form depends on the assumptions about the prediction errors (Diebold and Mariano, 2002).

## 3 Data

To understand which financial and non-financial features contribute more to the prediction of the cost of capital, we collect data from 2013 to 2019 for 1,433 publicly listed companies headquartered in 43 countries (the breakdown of sample composition according to country and territories is reported in the Supplementary Table 2).^1^

We select companies from the Index MSCI ACWI that includes large and mid-cap companies across 23 Developed Markets (DM) and 24 Emerging Markets (EM) countries, covering around 85% of the global investable equity opportunity set (https://www.msci.com/). Data and information are retrieved from the following sources: Refinitiv Eikon, I/B/E/S and Bloomberg for companies' financial and economic indicators; the World Bank, IMF and United Nation websites for country-level indicators.

Our dependent variable is the ex-ante cost of capital, derived from the forward earnings price ratio (Pinto, 2020) by computing the implicit return *r* for the company *i* according to Equation 10.


(10) $$\begin{array}{l} r_{\left(i,t\right)}=\frac{Earnings_{\left(i,t+1\right)}}{Price_{\left(i,t\right)}} \end{array}$$

As independent variables, we employ all the financial and non-financial variables individuated by the literature as relevant in the determination of the cost of capital, as well as country-specific features (Supplementary Table 1 in the Supplementary material lists and describes all variables used in the paper). We proxy financial information with key balance sheet and economic indicators. Additionally, we include non-financial performance using several ESG scores. The measures employed for ESG scores in empirical papers are not homogeneous (see the discussion by Agosto and Tanda, 2025). Previous studies commonly use measures obtained by commercial databases, such as Bloomberg or Refinitiv Eikon by Thomson Reuters (e.g., Breuer et al., 2018; Desender et al., 2020; Mariani et al., 2021; Wang et al., 2021; Tseng and Demirkan, 2021); other proxy are: the inclusion in sustainable/ESG indexes (e.g., Eom and Nam, 2017) or initiatives (Fisher-Vanden and Thorburn, 2011); own developed measures, sometimes based on previous literature (e.g., Michaels and Grúning, 2017; Lau, 2019); hybrid measures based on a mix of the above (e.g., Garćıa-Sánchez et al., 2021; Agosto et al., 2023). In this paper, we rely on Refinitiv Eikon and Bloomberg ESG information.

## 4 Results

At first, we split the available data set into an eighty per cent train set and a twenty per cent test set. Before training the XGBoost model, we use the GridSearchCV function from the sklearn Python package to determine the optimal hyperparameter settings: it results in a learning rate equal to 0.015; and a maximal depth, equal to 4. We then apply the XGBoost model to the training data set and apply the learned model to predict the response values (cost of capital values) in the test data set.

The XGBoost model performs rather well: the predicted average cost of capital in the test set is 6.44 % against an actual mean cost of 6.42%. Furthermore, the Root Mean Squared Error (RMSE) between the predicted and actual observations is equal to 3%, about half of the mean value, indicating a small variability of the errors.

**Figure 1:**
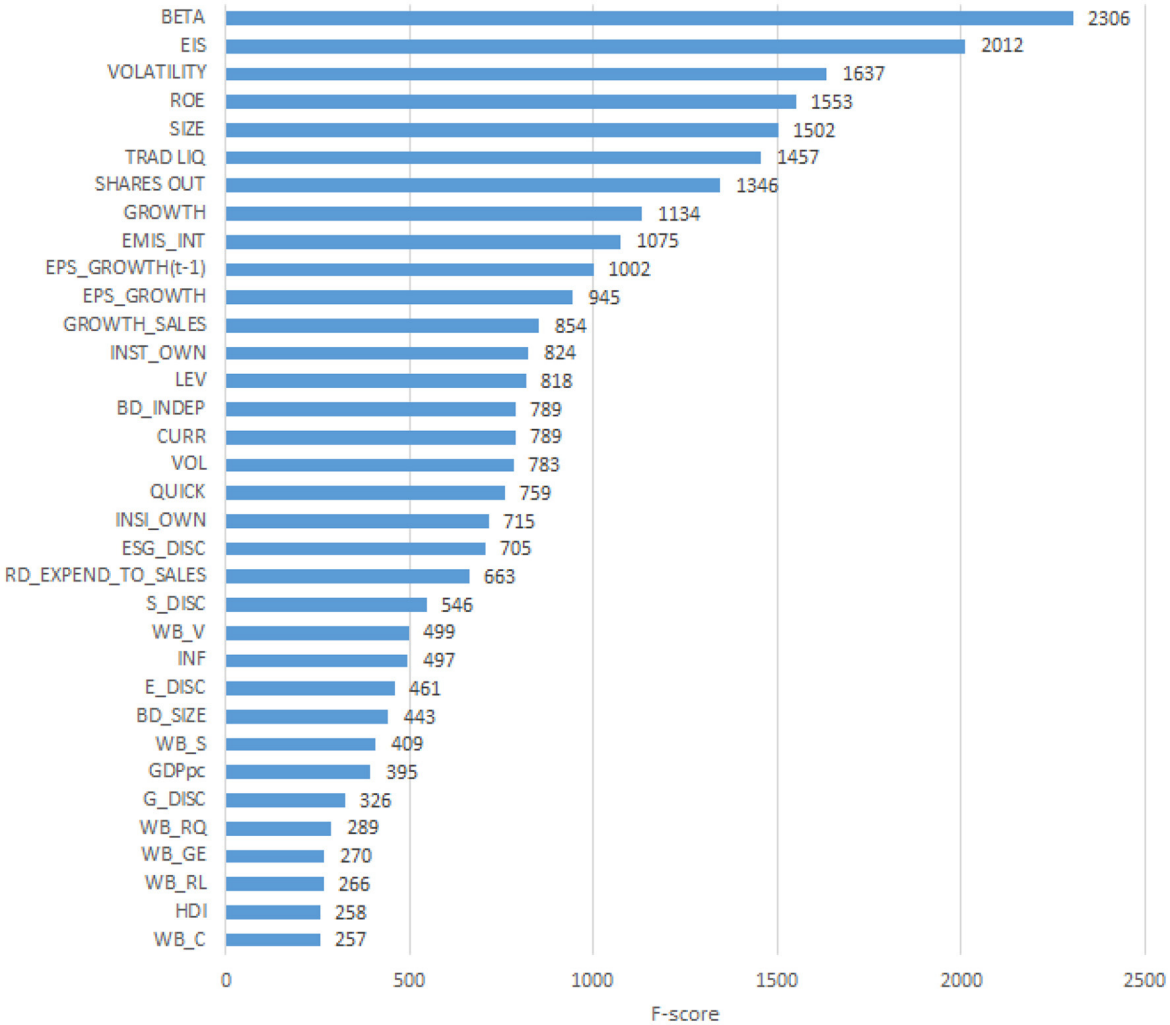
Feature importance plot. The Figure presents the feature importance plot computed using the XGBoost algorithm.

To explain the obtained predictions, we apply several different XAI methods. First, we analyse the results using the Feature Importance plot, based on the Gini Index, which is included into the XGBoost Python package. Figure 1 displays the results of the application.

**Figure 2:**
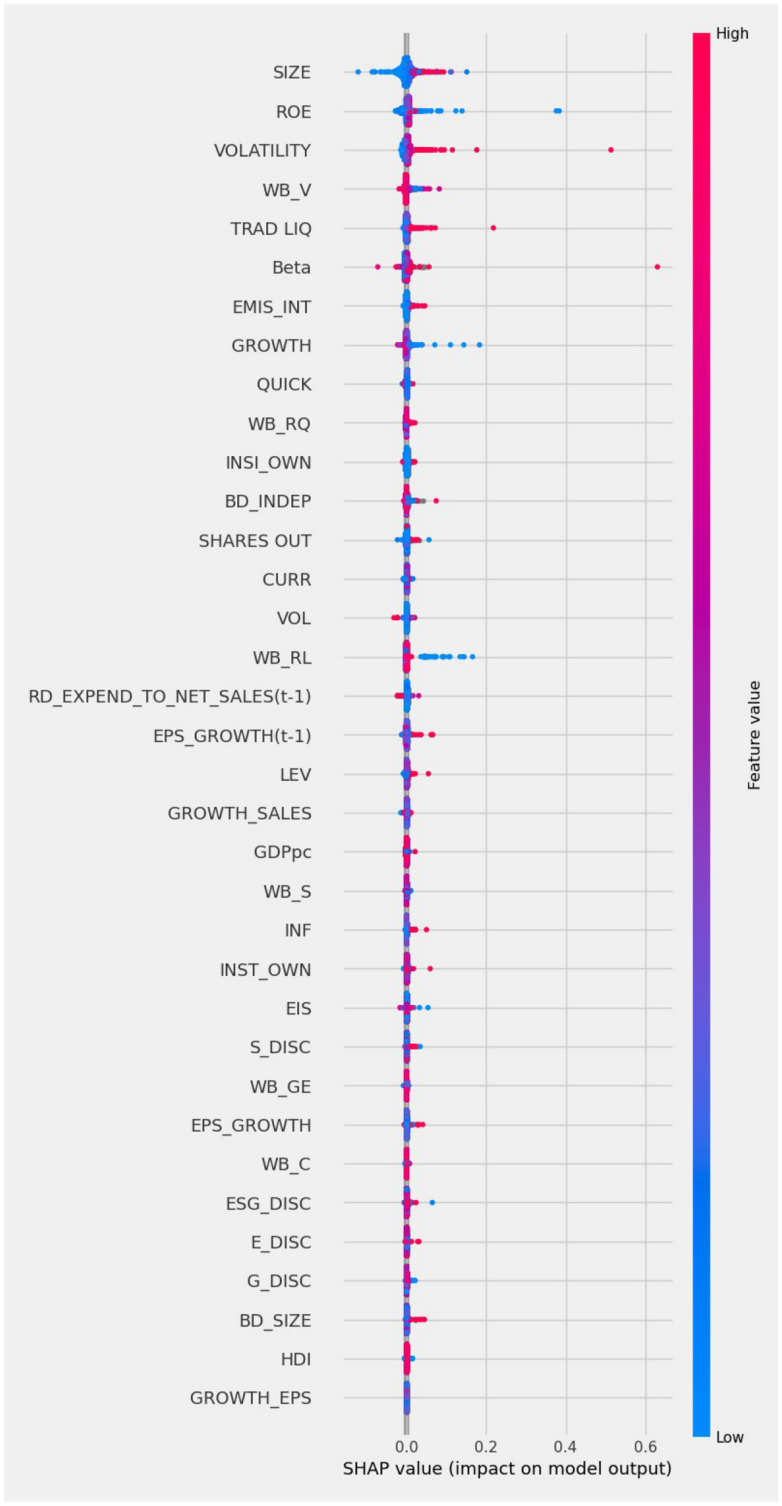
SHAP summary plot.

From Figure 1 we note that the five variables that rank the highest are, for each company, the systematic risk proxy (BETA), the environmental innovation score (EIS), the stock price volatility (VOLATILITY), the profitability measured as Return on Equity (ROE), and the size of the company (SIZE). However, it is well known that the feature importance plot is a component of tree models, whose results are not stable, as obtained on subsamples, and not globally (Altmann et al., 2010). To improve the robustness of the explanation, and overcome the weaknesses of the Feature importance plot, we analyse the same predictions using Shapley values. The calculated SHAP values can be visualised as a summary plot, as in Figure 2.

The SHAP summary plot shows the importance of the variables according to their contributions to the model predictions of the cost of capital. The variables are ordered according to their importance, from the most important (top) to the last important (bottom). In the Figure, each dot represents one observation of the underlying data set. When the dots of the variable are located at the right of the 0.000 vertical line it means that the variable has a positive impact on the prediction of the cost of capital; the opposite occurs when the dot is on the left. Blue shades of the dots represent low values of the underlying independent variable and red represents high values of the independent variable.

From Figure 2 we note that the variables contributing more to prediction are, for each company: the size (SIZE), the profitability measured as Return on Equity (ROE), the stock price volatility (VOLATILITY), country's voice—i.e., a variable which describes citizens' right to vote and freedom to convey opinions—(WB-V) and the liquidity of the company stocks traded (TRAD LIQ). Furthermore, we see that below the Beta, as a measure of the systematic risk of the companies' stock, the first firm's ESG performance indicators appears, namely the Greenhouse Gas (GHG) emissions intensity (EMIS_INT). Aside traditional financial indicators, also WB_RQ (country's regulatory quality) and the corporate governance characteristics (insider ownership – INSI_OWN — and the percentage of independent directors in the board – BD_indep) rank relatively high, and higher than trading volume (VOL), the firms' research and development effort (RD_EXPEND_TO_NET_SALES) and Earnings per share growth (EPS_GROWTH).

**Figure 3:**
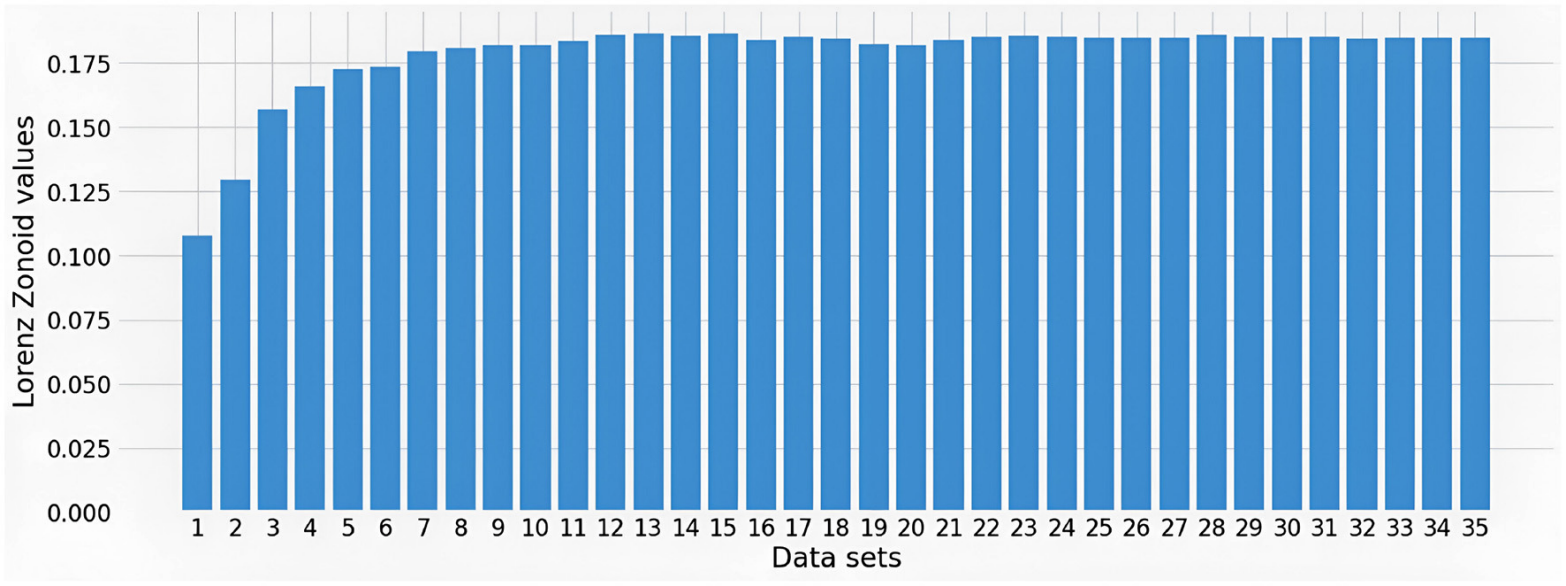
Lorenz Zonoid values plot.

Comparing the five most important variables in Figure 1 with those in Figure 2 note that three of them coincide, namely, size (SIZE), Return on Equity (ROE) and Stock price volatility (VOLATILITY). The systematic risk proxy Beta ranked first by XGBoost model in Figure 1 becomes sixth in the SHAP summary plot in Figure 2. Trade liquidity is sixth in Figure 1 and fifth in Figure 2. Other interesting differences include, for instance, the placement of the variable Environmental Innovation Score (EIS), that ranks second for XGBoost in Figure 1 and only 25th in Figure 2. Conversely, the country's institutional quality, namely the variable “Voice” (WB-V), is captured among the most important variables by Shapley values only. The difference between the two XAI tools may be due to the inclusion of many variables in the machine learning model, some of which have only a very small impact. This suggests performing a preliminary feature selection, to improve the robustness of the model. To this aim, we create a series of sub-datasets based on the feature ranking in the SHAP approach. The first data set consists only of the most important variable (SIZE). The second data set consists of the most important and the second most important variable (SIZE and ROE). We continue this subdivision until we obtain 35 sub-datasets, corresponding to all considered variables. We calculate the Lorenz Zonoid values for each of the chosen sub-datasets, which corresponds to an increasing number of explanatory variables: from 1 to 35. Figure 3 represents graphically the Lorenz Zonoid values calculated on each of the 35 subsets, ordered from the smallest (with only one variable included in the model) to the largest (all variables included in the model).

From Figure 3, we note that the highest Lorenz Zonoid value (0.1865) is achieved with the inclusion in the model of 13 variables. In other words, Figure 3 indicates that, according to the parsimony principle, good predictions are likely to be obtained by drastically simplifying the model from 35 to 13 features: a much simpler model.

Before concluding with the choice of a model with 13 variables, note that Figure 3 shows lower increments of Lorenz Zonoid values already after including four variables. When comparing the MSE of the model with 13 variables (0.0010) with the MSE of the model with 4 variables (0.0012), it can be seen that the model which includes 13 variables performs only slightly better.

The feature SIZE, which represents the asset size of a company, explains about 11% of the predictive accuracy of the model. When ROE is added to SIZE there is an increase of about 2% in accuracy. Adding VOLATILITY induces a further increase of about 2% and adding WB_V produces an increase of about 1%. To gain a better insight on whether to further simplify the chosen machine learning model, from 13 to four variables, we further analyse our results with the help of the Diebold Mariano test (Diebold and Mariano, 2002). More precisely, we compare the model which consists of only four variables with the model which consists of 11 variables, based on the results of the Lorenz Zonoid approach. The result of the test gives a *p*-value of 0.999. Since the *p*-value is higher than 0.05 the null hypothesis that the predictive power of the simpler model (with four variables) is as good as the predictive power of the more complex model (with eleven variables) cannot be rejected. Thus, the results of the Diebold Mariano test show that we can exclude all other variables from the data set and select a much simpler model that only contains four variables: SIZE, ROE, VOLATILITY, and WB_V.

From an economic viewpoint, the four chosen variables appear the most relevant in predicting the ex-ante cost of capital. Three of them refer to the well-known financial characteristics of a company. The fourth one is a non-financial country-related feature.

The variable SIZE is the most important variable for the XGBoost model and it represents the asset size of a company. Concerning the sign of importance, it can clearly be seen from the SHAP summary plot in Figure 2 that companies with a large asset size have a positive impact on the model's predictions of the cost of capital. Hence, the model predicts a higher cost of capital for companies with a large asset size and a lower cost of capital for companies with a small asset size. This seems to contradict the general idea that firms with bigger size can benefit from economies of scale (Stigler, 1958) than those with smaller asset sizes. Also according to asymmetries of information we would expect the opposite effect, with larger companies being less exposed to asymmetries of information and hence able to access to cheaper funding (Armstrong et al., 2011; Embong et al., 2012; He et al., 2013). Nevertheless, our sample includes all very large multinational listed companies. Hence, among this specific set of very large companies, size over a certain threshold can be perceived unable to pursue additional economies of scale; excessive size can also be interpreted as a factor contributing to complexity and opaqueness, therefore increasing perceived risk by the investors for these companies that become “too large” and “too complex.”

With reference to profitability, Figure 2 shows that low values of ROE have a strong impact on the models' predictions. High values of volatility of a company lead to increased predictions of the cost of capital. Unsurprisingly, investors associate a high volatility of a company stock price with higher risk and uncertainty and, as a consequence, a higher cost of capital.

As already mentioned, the fourth most important variable is WB_V, a country specific feature. The variable describes the political and regulatory framework of the country, describing how a country's citizens express their votes for the government and how their opinions are conveyed and heard. We can see from the SHAP summary plot in Figure 2 that high values of this variable have a strong impact on the models' predictions, leading to an increase in the predicted cost of capital.

We finally remark that our empirical findings using SHAP (Figure 2) indicate that the company's emissions are a significant predictor of the cost of capital, although the variable is not included in the selected parsimonious model with four variables by the Lorenz Model Selection approach. This result may be due to corporate emissions being related to important variables such as the size and ROE of a company, as well as the institutional quality of a country, described by variable WB_V.

## 5 Discussion

The discussion on the environmental, social, and governance performance of companies has become extremely important in the policy agenda among market investors and for corporates.

This paper contributes to the literature that evaluates the influence of non-financial factors in shaping firm's riskiness, here interpreted as ex-ante cost of capital. Although a number of studies investigates this issue, most of the empirical investigations make strong assumptions on the linearity of dependence between the cost of capital and its determinants. Previous papers, additionally, generally focus on financial determinants and, though more recently, on firms' non-financial characteristics, while only a few studies include country institutional settings (Desender et al., 2020; Wang et al., 2021; Yu et al., 2021). In this paper, we aim to fill this gap, by employing XAI methods to predict the cost of capital for a sample of multinational companies listed worldwide. To this end, among the determinants of cost of capital, we include standard financial proxy (e.g., profitability, company characteristics, market measures), firm non financial variables (e.g., ESG performance, carbon emissions) and country characteristics (e.g., institutional quality) that have been lately addressed as relevant in shaping companies' riskiness.

The findings of this study confirm the relevance of country and firms' non-financial features in determining the cost of capital for companies, interpreted in this paper as the perceived riskiness of investment. Indeed, the XAI methods employed rank among the most relevant variables predicting cost of capital not only traditional financial performance and market measures, but also country's institutional quality and firms' environmental performance.

These results have implications for firms and policymakers. Firms should individuate the drivers of the cost of capital and make appropriate actions to improve not only their financial profile but also their ESG performance, e.g., by reducing emission intensity or endowing the governance with effective tools that promote “good governance,” such as the presence of independent directors. Our study hence corroborate the increasing attention devoted by companies on ESG disclosure and sustainable behaviour (El Ghoul et al., 2011; Yu et al., 2018, 2021; De Giuli et al., 2024).

For policymakers our results show not only support of their efforts in regulating the disclosure of homogenous ESG data at the firm level (Agosto et al., 2023), but also the need to improve the country's institutional quality, to attract more investors and hence reduce the overall cost of funding for companies (Merton et al., 1987). Given the need for resources in the process of “greening the economy,” attracting less expensive funding is essential to finance investments in the corporate sector.

This paper has also limitations. Future research could employ alternative XAI methods and provide a comparison in terms of predictive accuracy, accounting also for the specific features of developed and developing countries. Also, as market liquidity appears to be a relevant factor, differentiating between efficient and inefficient markets is a further area that deserves investigation. Finally, as the regulatory provisions on the disclosure of ESG performance for companies evolve, behaviour of corporates might change and, also, investors' perceived risk can be driven by different factors.

## 6 Conclusions

This paper investigates for the first time the determinants of the cost of capital through a machine learning model, in combination with the SHAP framework and the Lorenz Zonoid approaches, to make it explainable. We are able to overcome the a priori hypothesis on the linearity of the relationship between variables and are able to individuate and rank the features that contribute more to the prediction of the cost of capital.

Overall, our results show that a firm's size, ROE, portfolio volatility risk, ESG behaviour and country's institutional quality are the relevant variables in predicting a firm's ex-ante cost of capital. With reference to non-financial features, the Shapley values approach shows that some of the non-financial indicators, proxied by ESG factors, such as Emission intensity or corporate governance settings, can be adopted as good predictors of the cost of equity besides the traditional financial features of companies. These results corroborate the proposals made by policymakers on the opportunity to disclose ESG performance of companies, including their GHG emissions (Agosto et al., 2023). Results suggest that the market penalises companies with high emission intensity, associated with more expensive capital funding. On the other hand, the market awards companies with good corporate governance practices by charging a lower cost of capital, e.g., the inclusion of independent directors.

Additional empirical results employing Lorenz Zonoid confirms that a firm's cost of capital is well predicted by a parsimonious model that includes the level of the country's voice, which we use to proxy the institutional quality of the country where the firm is incorporated.

In summary, our study provides supporting evidence that some key non-financial features both at firm and country level can contribute to shaping investors' risk perception and should be therefore included in companies' evaluation by investors. Future research can be devoted to understanding if and how these results change depending on the industries considered or over time, as regulation is modified and sustainability becomes integrated into the institutional setting of the different countries.

## Data Availability

The original contributions presented in the study are included in the article/Supplementary material, further inquiries can be directed to the corresponding author.

## Footnotes

^1^Around 500 companies are located in the US and slightly more than 200 in Japan. We rerun the XGBoost algorithm excluding these companies and the baseline results are generally confirmed.

## References

[B1] Adam C. (2024). Segmenting female students' perceptions about fintech using explainable AI. Front. Artif. Intell. 7:1504963. 10.3389/frai.2024.1504963 39726890 PMC11670257

[B2] Agosto A. Giudici P. Tanda A. (2023). How to combine ESG scores? a proposal based on credit rating prediction. Corp. Soc. Responsib. Environ. Manag. 30, 3222–3230. 10.1002/csr.2548

[B3] Agosto A. Tanda A. (2025). Divergence and aggregation of ESG ratings: a survey. Open Res. Eur. 5:28. 10.12688/openreseurope.19238.1 PMC12398680 40895218

[B4] Altmann A. Toloşi L. Sander O. Lengauer T. (2010). Permutation importance: a corrected feature importance measure. Bioinformatics 26, 1340–1347. 10.1093/bioinformatics/btq134 20385727

[B5] Armstrong C. S. Core J. E. Taylor D. J. Verrecchia R. E. (2011). When does information asymmetry affect the cost of capital? J. Acct. Res. 49, 1–40. 10.1111/j.1475-679X.2010.00391.x

[B6] Audemard G. Coste-Marquis S. Marquis P. Sabiri M. Szczepanski N. (2024). “Designing an XAI interface for tree-based ML models,” in The 27th European Conference on Artificial Intelligence (Santiago de Compostela). 10.3233/FAIA240599

[B7] Babaei G. Giudici P. Raffinetti E. (2025). A rank graduation box for SAFE AI. Expert Syst. Appl. 259:125239.

[B8] Banerjee R. Gupta K. Krishnamurti C. (2022). Does corrupt practice increase the implied cost of equity? J. Corp. Finance 73:102191. 10.1016/j.jcorpfin.2022.102191

[B9] Barg J. A. Drobetz W. El Ghoul S. Guedhami O. Schróder H. (2024). Institutional dual ownership and voluntary greenhouse gas emission disclosure. J. Corp. Finance 89:102671. 10.1016/j.jcorpfin.2024.102671

[B10] Basher S. A. Sadorsky P. (2025). How important are climate change risks for predicting clean energy stock prices? Evidence from machine learning predictive modeling and interpretation. J. Clim. Finance 10:100058. 10.1016/j.jclimf.2024.100058

[B11] Bentéjac C. Csórgő A. Martínez-Muñoz G. (2021). A comparative analysis of gradient boosting algorithms. Artif. Intell. Rev. 54, 1937–1967. 10.1007/s10462-020-09896-5

[B12] Bitetto A. Cerchiello P. Filomeni S. Tanda A. Tarantino B. (2023). Machine learning and credit risk: empirical evidence from small-and mid-sized businesses. Socioecon. Plann. Sci. 90:101746. 10.1016/j.seps.2023.101746

[B13] Breuer W. Múuller T. Rosenbach D. Salzmann A. (2018). Corporate social responsibility, investor protection, and cost of equity: a cross-country comparison. J. Bank. Finance 96, 34–55. 10.1016/j.jbankfin.2018.07.018

[B14] Bui B. Moses O. Houqe M. N. (2020). Carbon disclosure, emission intensity and cost of equity capital: multi-country evidence. Acct. Finance 60, 47–71. 10.1111/acfi.12492

[B15] Bussmann N. Giudici P. Marinelli D. Papenbrock J. (2020). Explainable AI in fintech risk management. Front. Artif. Intell. 3:26. 10.3389/frai.2020.00026 33733145 PMC7861223

[B16] Bussmann N. Giudici P. Marinelli D. Papenbrock J. (2021). Explainable machine learning in credit risk management. Comput. Econ. 57, 203–216. 10.1007/s10614-020-10042-0

[B17] Campbell J. L. (2007). Why would corporations behave in socially responsible ways? an institutional theory of corporate social responsibility. Acad. Manag. Rev. 32, 946–967. 10.5465/amr.2007.25275684

[B18] Cao L. (2022). AI in finance: challenges, techniques, and opportunities. ACM Comput. Surv. 55, 1–38. 10.1145/3502289

[B19] Černevicienė J. Kabasinskas A. (2024). Explainable artificial intelligence (XAI) in finance: a systematic literature review. Artif. Intell. Rev. 57:216. 10.1007/s10462-024-10854-8

[B20] Chen T. Guestrin C. (2016). “Xgboost: a scalable tree boosting system,” in Proceedings of the 22nd ACM SIGKDD International Conference on Knowledge Discovery and Data Mining (New York, NY: Association for Computing Machinery), 785–794. 10.1145/2939672.2939785

[B21] Dalwai T. Habib A. M. Mohammadi S. S. Hussainey K. (2023). Does managerial ability and auditor report readability affect corporate liquidity and cost of debt? Asian Rev. Acc. 31, 437–459. 10.1108/ARA-06-2022-0151

[B22] D'Amato D. Droste N. Allen B. Kettunen M. Láhtinen K. Korhonen J.. (2017). Green, circular, bio economy: a comparative analysis of sustainability avenues. J. Clean. Prod. 168, 716–734. 10.1016/j.jclepro.2017.09.053

[B23] De Giuli M. E. Grechi D. Tanda A. (2024). What do we know about esg and risk? a systematic and bibliometric review. Corp. Soc. Responsib. Environ. Manag. 31, 1096–1108. 10.1002/csr.2624

[B24] Desender K. Lóopez-Puertas M. Pattitoni P. Petracci B. (2020). Corporate social responsibility and cost of financing-The importance of the international corporate governance system. Wiley 28, 187–273. 10.1111/corg.12312

[B25] Diebold F. X. Mariano R. S. (2002). Comparing predictive accuracy. J. Bus. Econ. Stat. 20, 134–144. 10.1198/07350010275341044412611515

[B26] Dorfleitner G. Halbritter G. Nguyen M. (2015). Measuring the level and risk of corporate responsibility-an empirical comparison of different ESG rating approaches. J. Asset Manag. 16, 450–466. 10.1057/jam.2015.31

[B27] El Ghoul S. Guedhami O. Kwok C. C. Mishra D. R. (2011). Does corporate social responsibility affect the cost of capital? J. Bank. Finance 35, 2388–2406. 10.1016/j.jbankfin.2011.02.007

[B28] Eldomiaty T. I. Al Qassemi T. B. F. Mabrouk A. F. Abdelghany L. S. (2016). Institutional quality, economic freedom and stock market volatility in the MENA region. Macroecon. Finance Emerg. Mark. Economies 9, 262–283. 10.1080/17520843.2015.1093011

[B29] Embong Z. Mohd-Saleh N. Sabri Hassan M. (2012). Firm size, disclosure and cost of equity capital. Asian Rev. Acc. 20, 119–139. 10.1108/13217341211242178

[B30] Eom K. Nam G. (2017). Effect of entry into socially responsible investment index on cost of equity and firm value. Sustainability 9:717. 10.3390/su9050717

[B31] Feng S. Wareewanich T. (2024). The effects of corporate carbon performance on financing cost-evidence from S&P 500. J. Infrastr. Policy Dev. 8:7997. 10.24294/jipd.v8i10.7997

[B32] Fisher-Vanden K. Thorburn K. S. (2011). Voluntary corporate environmental initiatives and shareholder wealth. J. Environ. Econ. Manag. 62, 430–445. 10.1016/j.jeem.2011.04.003

[B33] Fryer D. Strúmke I. Nguyen H. (2021). Shapley values for feature selection: the good, the bad, and the axioms. IEEE Access 9, 144352–144360. 10.1109/ACCESS.2021.3119110

[B34] Gan L. Wang H. Yang Z. (2020). Machine learning solutions to challenges in finance: an application to the pricing of financial products. Technol. Forecast. Soc. Change 153:119928. 10.1016/j.techfore.2020.119928

[B35] García-Sánchez I.-M. Hussain N. Khan S.-A. Martínez-Ferrero J. (2021). Do markets punish or reward corporate social responsibility decoupling? Bus. Soc. 60, 1431–1467. 10.1177/0007650319898839

[B36] Garriga E. Melé D. (2004). Corporate social responsibility theories: mapping the territory. J. Bus. Ethics 53, 51–71. 10.1023/B:BUSI.0000039399.90587.34

[B37] Ghoddusi H. Creamer G. G. Rafizadeh N. (2019). Machine learning in energy economics and finance: a review. Energy Econ. 81, 709–727. 10.1016/j.eneco.2019.05.00636628421

[B38] Giudici P. Raffinetti E. (2020). Lorenz model selection. J. Classif. 37, 754–768. 10.1007/s00357-019-09358-w

[B39] Global Sustainable Investment Alliance. (2018). 2018 Global Sustainable Investment Review. Brussels.

[B40] Gramegna A. Giudici P. (2021). SHAP and LIME: an evaluation of discriminative power in credit risk. Front. Artif. Intell. 4:752558. 10.3389/frai.2021.752558 34604738 PMC8484963

[B41] Gramegna A. Giudici P. (2022). Shapley feature selection. FinTech 1, 72–80. 10.3390/fintech1010006

[B42] Grira J. Hassan M. K. Labidi C. Soumaré I. (2019). Equity pricing in Islamic banks: international evidence. Emerg. Mark. Finance Trade 55, 613–633. 10.1080/1540496X.2018.1451323

[B43] Gupta K. (2018). Environmental sustainability and implied cost of equity: international evidence. J. Bus. Ethics 147, 343–365. 10.1007/s10551-015-2971-z

[B44] Hail L. Leuz C. (2006). International differences in the cost of equity capital: do legal institutions and securities regulation matter? J. Acct. Res. 44, 485–531. 10.1111/j.1475-679X.2006.00209.x

[B45] He W. P. Lepone A. Leung H. (2013). Information asymmetry and the cost of equity capital. Int. Rev. Econ. Finance 27, 611–620. 10.1016/j.iref.2013.03.001

[B46] Houqe M. N. Khan H. Z. Moses O. Elias A. (2024). Corporate reputation, cost of capital and the moderating role of economic development: international evidence. Medit. Account. Res. 32, 1106–1134. 10.1108/MEDAR-03-2023-1951

[B47] Huang H. H. Wang C. Xie H. Zhou J. (2021). Independent director attention and the cost of equity capital. J. Bus. Finance Account. 48, 1468–1493. 10.1111/jbfa.12522

[B48] Jensen M. C. Meckling W. H. (1976). Theory of the firm: managerial behavior, agency costs and ownership structure. J. Finan. Econ. 3, 305–360. 10.1016/0304-405X(76)90026-X

[B49] Koshevoy G. (1995). Multivariate Lorenz majorization. Soc. Choice Welfare 12, 93–102. 10.1007/BF00182196

[B50] Kumar I. E. Venkatasubramanian S. Scheidegger C. Friedler S. (2020). “Problems with shapley-value-based explanations as feature importance measures,” in International Conference on Machine Learning (PMLR), 5491–5500.

[B51] Kumar R. Gupta P. Bhawna (2025). “Application of explainable artificial intelligence in fintech,” *Generative Artificial Intelligence in Finance: Large Language Models, Interfaces, and Industry Use Cases to Transform Accounting and Finance Processes* (Hoboken, NJ), 383–405. 10.1002/9781394271078.ch19

[B52] Lau C. K. (2019). The economic consequences of business sustainability initiatives. Asia Pac. J. Manag. 36, 937–970. 10.1007/s10490-018-9623-7

[B53] Lerman R. I. Yitzhaki S. (1984). A note on the calculation and interpretation of the Gini index. Econ. Lett. 15, 363–368. 10.1016/0165-1765(84)90126-5

[B54] Lin B. Bai R. (2022). Machine learning approaches for explaining determinants of the debt financing in heavy-polluting enterprises. Financ. Res. Lett. 44:102094. 10.1016/j.frl.2021.102094

[B55] Lin J. (2018). Using weighted shapley values to measure the systemic risk of interconnected banks. Pac. Econ. Rev. 23, 244–270. 10.1111/1468-0106.12155

[B56] Liu L. Chen C. Wang B. (2022). Predicting financial crises with machine learning methods. J. Forecasting 41, 871–910. 10.1002/for.2840

[B57] Lundberg S. M. Erion G. Chen H. DeGrave A. Prutkin J. M. Nair B.. (2020). From local explanations to global understanding with explainable AI for trees. Nat. Mach. Intell. 2, 56–67. 10.1038/s42256-019-0138-9 32607472 PMC7326367

[B58] Lundberg S. M. Lee S.-I. (2017). “A unified approach to interpreting model predictions,” in 31st Conference on Neural Information Processing Systems (NIPS 2017) (Long Beach, CA), 30.

[B59] Mariani M. Pizzutilo F. Caragnano A. Zito M. (2021). Does it pay to be environmentally responsible? Investigating the effect on the weighted average cost of capital. Corp. Soc. Responsib. Environ. Manag. 28, 1854–1869. 10.1002/csr.2164

[B60] Merton R. C. (1987). A simple model of capital market equilibrium with incomplete information. J. Finance 42, 483–510. 10.2307/232836726825754

[B61] Michaels A. Grúning M. (2017). Relationship of corporate social responsibility disclosure on information asymmetry and the cost of capital. J. Manag. Control 28, 251–274. 10.1007/s00187-017-0251-z

[B62] Mosler K. (1994). Majorization in economic disparity measures. Lin. Alg. Appl. 199, 91–114. 10.1016/0024-3795(94)90343-3

[B63] Nasrallah N. El Khoury R. Atayah O. F. Marashdeh H. Najaf K. (2025). The impact of carbon awareness, country-governance, and innovation on the cost of equity: evidence from oil and gas firms. Res. Int. Bus. Finance 73:102640. 10.1016/j.ribaf.2024.102640

[B64] Ortmann K. M. (2016). The link between the Shapley value and the beta factor. Decis. Econ. Finance 39, 311–325. 10.1007/s10203-016-0178-0

[B65] Pástor L. Sinha M. Swaminathan B. (2008). Estimating the intertemporal risk-return tradeoff using the implied cost of capital. J. Finance 63, 2859–2897. 10.1111/j.1540-6261.2008.01415.x

[B66] Pinto J. E. (2020). Equity Asset Valuation. CFA Institute Investment Series. Hoboken, NJ: John Wiley & Sons.

[B67] Polak P. Nelischer C. Guo H. Robertson D. C. (2020). “Intelligent” finance and treasury management: what we can expect. AI Soc. 35, 715–726. 10.1007/s00146-019-00919-6

[B68] Ross S. A. (1973). The economic theory of agency: the principal's problem. Am. Econ. Rev. 63, 134–139.

[B69] Sarang A. A. A. Aubert N. Hollandts X. (2024a). Board gender diversity and the cost of equity: what difference does gender quota legislation make? Int. J. Finance Econ. 29, 2193–2213. 10.1002/ijfe.2774

[B70] Sarang A. A. A. Rind A. A. Al-Faryan M. A. S. Saeed A. (2024b). Women on board and the cost of equity: the mediating role of information asymmetry. J. Finan. Report. Account. 22, 1356–1379. 10.1108/JFRA-02-2022-0048

[B71] Shad M. K. Lai F.-W. Shamim A. McShane M. (2020). The efficacy of sustainability reporting towards cost of debt and equity reduction. Environ. Sci. Pollut. Res. 27, 22511–22522. 10.1007/s11356-020-08398-9 32319056

[B72] Shalit H. (2023). Weighted shapley values of efficient portfolios. Risk Decis. Anal. 9, 31–38. 10.3233/RDA-231507

[B73] Shapley L. S. (1953). Stochastic games. Proc. Natl. Acad. Sci. 39, 1095–1100. 10.1073/pnas.39.10.1953 16589380 PMC1063912

[B74] Sharma S. Vredenburg H. (1998). Proactive corporate environmental strategy and the development of competitively valuable organizational capabilities. Strateg. Manag. J. 19, 729–753

[B75] Simonian J. (2019). Portfolio selection: a game-theoretic approach. J. Portf. Manag. 45, 108–116. 10.3905/jpm.2019.1.095

[B76] Stigler G. J. (1958). The economies of scale. J. Law Econ. 1, 54–71. 10.1086/466541

[B77] Surroca J. Tribó J. A. Waddock S. (2010). Corporate responsibility and financial performance: the role of intangible resources. Strateg. Manag. J. 31, 463–490. 10.1002/smj.820

[B78] Tran Q. T. (2020). Ownership structure and demand for independent directors: evidence from an emerging market. J. Econ. Dev. 22, 335–342. 10.1108/JED-03-2020-0022

[B79] Tron A. Dallocchio M. Ferri S. Colantoni F. (2023). Corporate governance and financial distress: lessons learned from an unconventional approach. J. Manag. Gov. 27, 425–456. 10.1007/s10997-022-09643-8

[B80] Tseng C.-Y. Demirkan S. (2021). Joint effect of CEO overconfidence and corporate social responsibility discretion on cost of equity capital. J. Contemp. Account. Econ. 17:100241. 10.1016/j.jcae.2020.100241

[B81] UNDP. (2018). Human Development Index. New York, NY: United Nations Development Programme.

[B82] Wang K. T. Kartika F. Wang W. W. Luo G. (2021). Corporate social responsibility, investor protection, and the cost of equity: evidence from east asia. Emerg. Mark. Rev. 47:100801. 10.1016/j.ememar.2021.100801

[B83] Weber P. Carl K. V. Hinz O. (2024). Applications of explainable artificial intelligence in finance–a systematic review of finance, information systems, and computer science literature. Manag. Rev. Q. 74, 867–907. 10.1007/s11301-023-00320-0

[B84] Widyawati L. (2020). A systematic literature review of socially responsible investment and environmental social governance metrics. Bus. Strat. Environ. 29, 619–637. 10.1002/bse.239336969097

[B85] Wong W. C. Batten J. A. Mohamed-Arshad S. B. Nordin S. Adzis A. A.. (2021). Does ESG certification add firm value? Financ. Res. Lett. 39:101593. 10.1016/j.frl.2020.101593

[B86] World Bank (2018). Worldwide Governance Indicators. The World Bank. Available online at: www.govindicators.org (accessed October 30, 2024).

[B87] Yu E. P.-Y. Guo C. Q. Luu B. V. (2018). Environmental, social and governance transparency and firm value. Bus. Strat. Environ. 27, 987–1004. 10.1002/bse.204734607134

[B88] Yu E. P.-Y. Tanda A. Luu B. V. Chai D. H. (2021). Environmental transparency and investors' risk perception: cross-country evidence on multinational corporations' sustainability practices and cost of equity. Bus. Strat. Environ. 30, 3975–4000. 10.1002/bse.2852

[B89] Zimon G. Habib A. M. Haluza D. (2024). Does the quality management system affect working capital management efficiency? evidence from polish firms. Cogent Bus. Manag. 11:2292787. 10.1080/23311975.2023.2292787

