# Supplementary material for: Explainable machine learning to predict the cost of capital
Source: Front Artif Intell. 2025 Apr 10;8:1578190. doi: 10.3389/frai.2025.1578190 (PMC12018374; doi:10.3389/frai.2025.1578190)
Supplement: Supplementary file 1 [file Supplementary_file_1.pdf]

# Explainable Machine Learning to predict the Cost of Capital

**Niklas Bussmann** [1], **Paolo Giudici** [1,2], **Alessandra Tanda** [1,2,*] **and Ellen Pei-Yi Yu** [3]

[1] *Department of Economics and Management, University of Pavia, Pavia, Italy*
[2] *CAM-Risk - Centre for the Analysis and Measurement of Global Risks, University of Pavia, Pavia, Italy*
[3] *Department of Management, Birkbeck College, University of London, London, UK*

Correspondence*:
Alessandra Tanda, Department of Economics and Management, University of Pavia,
Via San Felice al Monastero, 5 - 27100 Pavia, Italy
Corresponding Author
alessandra.tanda@unipv.it

# SUPPLEMENTARY MATERIALS

## Data availability statement

Data employed in this study are retrieved from commercial databases and are used under licence.

## Tables

Table 1 The considered explanatory variables

| Variable name | Variable description |
|---|---|
| Firm's financial features | *(source: Refinitiv Eikon, Bloomberg, I/B/E/S)* |
| SIZE | Value of a company's asset size. |
| ROE | The ratio of net income to 'shareholders' equity (Return on Equity). |
| VOLATILITY | Volatility of a company's stock price. |
| Beta | Beta is a proxy of systematic risk and shows how shares price move according to movements of the relative market index. |
| CURR | Current ratio: Indicator for a company's liquidity. |
| QUICK | Quick ratio: Indicator for a company's liquidity. |
| EPS-GROWTH(t-1) | Value of a company's growth rate on earnings per share (EPS) in t-1. |
| EPS-GROWTH | Value of a company's growth rate on earnings per share (EPS). |
| TRAD LIQ | Trading liquidity. |
| GROWTH SALES 1 | A company's growth rate on sales, based on a ratio of variations in sales over the previous year. |
| GROWTH SALES 3 | Value of a company's average annual growth rate on sales over the previous three years. |
| RD EXPEND TO NET SALES | Amount of Research and Development expenses divided by net sales. |
| LEV | Leverage: A ratio of a company's total debt to total assets. |
| VOL | The trading volume is used for calculating the volume-weighted average price. |
| SHARES OUT | A company's outstanding shares available on the market. |
| GROWTH-EPS | Value of a company's growth rate on earnings per share (EPS). |
| Firm's non-financial features | *(source: Refinitiv Eikon, Bloomberg, I/B/E/S)* |
| E-DISC | Bloomberg environmental disclosure score measures the amount of environmental information a company reveals to the public. If companies provide all data points collected by Bloomberg, the maximum value of 100 is awarded. The minimum value starts from 0.1. |

| | |
|---|---|
| G-DISC | Bloomberg governance disclosure score measures the amount of governance information a company reveals to the public. If companies provide all data points collected by Bloomberg, the maximum value of 100 is awarded. The minimum value starts from 0.1. |
| ESG-DISC | Bloomberg ESG disclosure score measures the amount of environmental, social, and governance information a company reveals to the public. If companies provide all data points collected by Bloomberg, the maximum value of 100 is awarded. The minimum value starts from 0.1. |
| S-DISC | Bloomberg social disclosure score measures the amount of social information a company reveals to the public. If companies provide all data points collected by Bloomberg, the maximum value of 100 is awarded. The minimum value starts from 0.1. |
| EMIS-INT | Emission intensity of a company. |
| EIS | Environmental Innovation Score (EIS) is a company's environmental innovation degree, measured based on a company's green revenue and its research and development expenses. |
| INSI-OWN | Insider ownership: a percentage of equities held by insiders. |
| BD-SIZE | The number of board members. |
| BD-INDEP | A percentage of the independent directors in a company's board. |
| INST-OWN | A percentage of equities held by a company's institutional investors. |
| Country non-financial features | *(Source: World Bank)* |
| WB-V | Indicator of voice for country $i$ represents observations of how a country's inhabitants have the right to vote for their government and freedom to convey their opinions. |
| WB-RQ | Indicator of the regulatory quality for country $i$ represents opinions on how a government implements its prudent policies to help the private sector grow. |
| WB-RL | Indicator of the rule of law for country $i$ represents perceptions of how agents have confidence in the general public rules. |
| WB-GE | Indicator of the government effectiveness for country $i$ represents the quality of a country's public service and a government's creditability to the public. |
| WB-C | Indicator of control of corruption for country $i$ represents observations of the degree to which the elite and the public power pursue their private interests. |
| WB-S | Indicator of political stability for country $i$ represents perceptions of the prospects of political uncertainty and terrorism. |
| HDI | The human development index quantifies a country's development in these key dimensions: its education, health, and economic aspect. |
| Other control variables | *(Source: IMF World Economic Outlook Database)* |

| | |
|---|---|
| INF | We collect the inflation rate for our sample countries from the IMF World Economic Outlook Database. |
| GDPpc | Log (GDP per capita) is measured based on the purchasing power parity exchange rates in 2011. Unit: the U.S. dollar. |

**Table 2.** Sample composition – countries and territories.

| Country or territory | number of firms | Country or territory | number of firms |
|---|---|---|---|
| Australia | 43 | Malaysia | 3 |
| Austria | 3 | Mexico | 12 |
| Belgium | 7 | Netherlands | 10 |
| Brazil | 37 | New Zealand | 5 |
| Britain | 63 | Norway | 8 |
| Canada | 1 | Peru | 1 |
| China | 80 | Philippines | 15 |
| Chile | 6 | Poland | 11 |
| Colombia | 3 | Portugal | 3 |
| Denmark | 15 | Qatar | 1 |
| Finland | 11 | Russia | 9 |
| France | 53 | Singapore | 18 |
| Germany | 48 | South Africa | 32 |
| Hong Kong | 62 | South Korea | 6 |
| Hungary | 2 | Spain | 14 |
| India | 3 | Sweden | 21 |
| Indonesia | 21 | Switzerland | 25 |
| Ireland | 5 | Taiwan | 6 |
| Israel | 4 | Thailand | 23 |
| Italy | 10 | Turkey | 13 |
| Japan | 207 | United States | 508 |
| Jordan | 1 | | |
| Luxembourg | 4 | **Total** | **1433** |
